# Decoding green space supply–demand mismatch through urban morphology: Toward equitable urban planning with explainable machine learning

**DOI:** 10.1371/journal.pone.0342596

**Published:** 2026-03-18

**Authors:** Lijuan Sun, Wei Liu, Qiqi Liu

**Affiliations:** 1 College of Horticulture, Jinling Institute of Technology, Nanjing, Jiangsu Province, People’s Republic of China,; 2 College of Landscape Architecture, Nanjing Forestry University, Nanjing, Jiangsu Province, People’s Republic of China; The Chinese University of Hong Kong, HONG KONG

## Abstract

Urban green spaces (UGS), as essential components of urban ecological infrastructure, play a vital role in improving residents’ quality of life and fostering spatial equity. However, rapid urbanization and intensive land use have intensified UGS supply–demand mismatches and associated “green inequities”, while their spatial drivers remain insufficiently understood. Taking the central urban area of Nanjing as a case study, this study developed a comprehensive framework to quantify UGS supply, demand, and spatial mismatches in 2022, thereby revealing inequities in green resource allocation. An explainable machine learning model (XGBoost–SHAP) was further applied to identify the urban morphological drivers of these mismatches. The results showed that all traffic analysis zones (TAZs) experienced varying degrees of spatial mismatch: 48 TAZs in urban core suffered from severe green space deficits, whereas 203 TAZs in peripheral areas exhibited evident surpluses. We found a clear gap between provision and residents’ actual benefits, with “quantitative abundance” not translating into “qualitative accessibility”. Regarding driving mechanisms, urban compactness and floor area ratio exerted significant negative impacts on supply–demand balance, while road density and land-use mix further amplified spatial inequities. In contrast, public facility coverage and the proportion of residential land played positive roles in mitigating mismatches. Based on these findings, we propose governance strategies that move beyond an “area-oriented” approach, emphasizing urban morphology optimization to align green space services with residents’ needs. Overall, this study offers practical insights for equity-oriented green space planning and spatial governance in rapidly urbanizing regions, advancing social equity and environmental justice.

## Introduction

Urban green spaces (UGS) provide essential ecological functions and public services that are critical for environmental quality, human well-being [1,2]. However, rapid urbanization and intensive land development have increasingly widened the gap between UGS supply and residents’ actual demand [3]. This mismatch has resulted in marked disparities in accessibility, service quality, and opportunities for use, reinforcing inequalities in spatial access to green resources [4]. Therefore, systematically assessing the UGS supply–demand relationship is essential for revealing its spatial patterns and equity implications.

Extensive studies have been conducted on the supply and demand of UGS. On the supply side, research has traditionally assessed green space quantity and spatial distribution using measures such as green coverage or per capita area [5]. Increasingly, studies also evaluate green space quality and service capacity, providing a more comprehensive view of supply [6]. On the demand side, research explores residents’ mobility patterns and accessibility constraints [7]. It highlights their reliance on walkable green spaces [8]. In addition, demographic characteristics, spatial accessibility, and activity patterns are often integrated to better capture actual demand [9,8]. With advancing research, scholars have widely recognized that many cities continue to face insufficient UGS supply alongside growing resident demand [10]. Consequently, examining UGS from a single supply- or demand-side perspective is no longer adequate for explaining observed utilization patterns. This limitation underscores the need for an integrated supply–demand framework to identify mismatches between the two [11]. However, research on how these mismatches form and the spatial factors that shape them is still limited.

The mismatch between the supply and demand of UGS is strongly shaped by urban morphology [12]. Different urban forms restructure population distribution and the built environment, thereby influencing residential patterns and daily mobility behaviors [13,14]. These morphological features interact to produce distinct spatial configurations of UGS supply and demand [15]. Therefore, analyzing UGS mismatches through the lens of urban morphology is crucial for identifying the underlying causes of uneven green space provision. This perspective further provides a scientific basis for optimizing green space layouts and enhancing public space provision [16]. However, when dealing with high-dimensional and multi-source urban data, traditional linear models are often constrained by multicollinearity and limited model fit, making it difficult to capture the nonlinear characteristics and threshold effects that are prevalent in practice [17]. Consequently, research on the nonlinear influences of urban morphology on UGS supply–demand mismatches and their key thresholds remains inadequate.

To address these gaps, this study examine the central urban area of Nanjing as a case study to quantify UGS supply–demand mismatches and reveal their spatial patterns. An explainable machine-learning model (XGBoost–SHAP) is then applied to identify the key morphological drivers and their threshold effects. The study focuse on two core questions: (1) What are the characteristics and spatial distribution of UGS supply–demand mismatches? (2) How are these mismatches associated with urban morphological factors? The findings can provide practical insights for urban planning and contribute to the development of more equitable green space systems.

## Methods

### Study area

**Fig 1:**
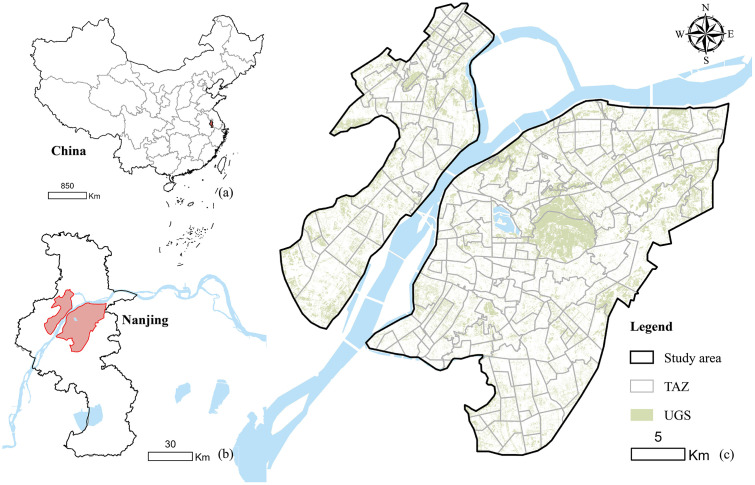
Location of the study area. **(a)** Geographical location of Nanjing within China. **(b)** Location of the study area within Nanjing. **(c)** TAZ and UGS in the study area. Base map and data from OpenStreetMap and OpenStreetMap Foundation.

Nanjing, located in the lower Yangtze River region of eastern China (31°14′–32°37′ N, 118°22′–119°14′ E), has a north subtropical monsoon climate with four distinct seasons and abundant rainfall, providing favorable conditions for UGS development. This study focuses on the central urban area of Nanjing, covering both the core districts and the surrounding expansion zones (Fig 1). This study area was selected because rapid urbanization has produced a complex spatial structure and a diverse population composition, providing a meaningful context for analyzing UGS-related spatial processes. Newly developed districts are generally well-equipped with public facilities and green spaces, while older residential communities and urban villages accommodate more disadvantaged groups and often experience insufficient green space provision. These pronounced contrasts in urban development patterns and service levels have resulted in clear spatial disparities in UGS distribution. Moreover, the evident imbalance between green space supply and residents’ needs makes this area a representative and policy-relevant setting for examining UGS supply–demand mismatch. As a rapidly urbanizing city, Nanjing reflects development patterns, population stratification, and green space governance structures that are common to many metropolitan areas. In addition, the availability of comprehensive spatial, demographic, and planning datasets supports systematic and robust empirical analysis, thereby strengthening the generalizability and transferability of the findings.

For spatial analysis, we adopted traffic analysis zone (TAZ), delineated by primary and secondary roads, as the basic analytical unit. The TAZ framework reflects residents’ travel behavior and ensures data operability, enabling neighborhood-scale assessment of green space supply, demand, and spatial mismatches [17]. A total of 251 TAZs were delineated, with an average area of 3.2 km². The TAZ system captures neighborhood-scale spatial variation, reflects typical patterns of residents’ travel behavior, and provides an appropriate spatial unit for analyzing UGS supply, demand, and supply–demand mismatch.

### Datasets

The datasets used in this study are summarized in Table 1. Specifically, The UGS information was obtained from the *Nanjing Municipal Bureau*, and POI datasets provided key urban functional attributes such as residential areas, schools, and hospitals. All datasets correspond to the year 2022.

**Table 1:** Data source and description.

Data	Source	Time	Description	TAZ-level mapping method	Indicator category
**Land cover**	Earth System Science Data (https://essd.copernicus.org/) [18]	2022	Raster, 1m	Zonal statistics	Natural environment
**Population density**	WorldPop (https://www.worldpop.org)	2022	Raster, 100m	Zonal statistics	Socioeconomic
**POI**	Amap (https://www.amap.com)	2022	Vector	Zonal statistics	Built environment
**AOI**	Amap (https://www.amap.com/)	2022	Vector	Spatial overlay	Built environment
**Building**	OpenStreetMap (https://www.openstreetmap.org)	2022	Vector	Spatial overlay	Built environment
**Road**	OpenStreetMap (https://www.openstreetmap.org)	2022	Vector	Spatial overlay	Built environment
**Per capita GDP**	*Nanjing Statistical Yearbook*	2022	–	Direct assignment	Socioeconomic

### Research framework

**Fig 2:**
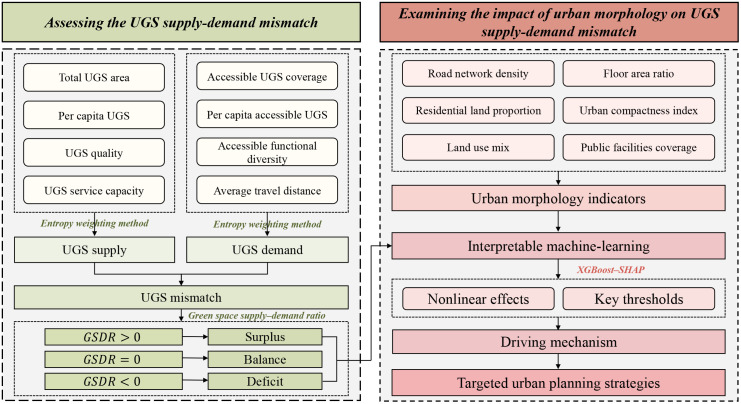
Research framework of study.

The research framework consisted of two main steps (Fig 2). The first step assessed the UGS supply–demand mismatch. Composite supply and demand indices were constructed using the entropy weighting method, and the supply–demand ratio was subsequently applied to delineate the mismatch status of each TAZ. The second step examined how urban morphological factors affected the UGS supply–demand mismatch. Using an interpretable machine-learning framework based on XGBoost–SHAP, the nonlinear effects and key thresholds of multiple morphological factors were systematically evaluated. Based on the identified mechanisms, targeted urban planning strategies were proposed for different types of urban areas to improve the balance between UGS supply and demand and support more equitable urban planning.

### Assessment of UGS supply–demand mismatch

#### Perspectives on UGS supply and demand.

**Fig 3:**
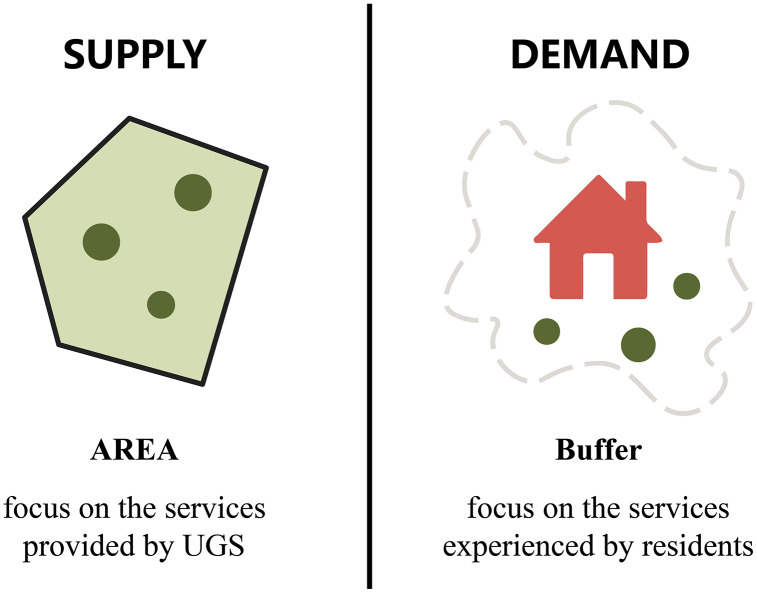
Perspectives on UGS supply and demand. **(a)** Supply side. **(b)** Demand side.

The relationship between UGS supply and demand can be conceptualized from two perspectives (Fig 3). The supply side focuses on “how much service green spaces can provide”, emphasizing the overall capacity of UGS based on its quantity, quality, and spatial distribution [19]. In this study, UGS supply was assessed at the TAZ level to capture regional provisioning capacity. In contrast, the demand side emphasizes “the services that residents can actually obtain and experience from green spaces”, reflecting the potential use of UGS from a resident-centered perspective [20]. Accordingly, UGS demand was evaluated within the 10-minute living circle, which represents the commonly accepted walking distance in daily life [21].

#### Evaluation of UGS supply.

On the supply side, the emphasis lies in assessing how much service green spaces can provide. Accordingly, we developed an indicator system that captures supply from multiple aspects (Table 2). Total UGS area represents the most direct measure of supply, reflecting the overall spatial extent available for ecological regulation and recreational use within each unit, and forming the basis of supply capacity [22]. Per capita UGS incorporates population factors to reveal differences in supply pressure and spatial equity, addressing the limitation that area-based indicators alone may obscure shortages in densely populated areas [23]. UGS quality highlights ecological performance and user experience, where higher vegetation coverage and healthier ecological conditions indicate greater environmental regulation and aesthetic value [24]. UGS service capacity reflects the effective service radius of green spaces, measuring their actual ability to serve surrounding residents [25]. Together, these indicators provide a comprehensive evaluation of UGS supply capacity. All spatial analyses of UGS supply were conducted using ArcGIS Pro 3.3 to ensure accurate spatial processing and visualization.

**Table 2:** Supply-side indicators.

Indicator	Direction	Description
**Total UGS area**	Positive	Total green space area within each TAZ. $\begin{array}{c} TUA=\sum_{i=\text{1}}^{m}A_{i} \end{array}$ where $A_{i}$ denotes the area of the i-th green space patch, and m is the total number of green space patches within the unit.
**Per capita UGS**	Positive	Green space area per resident. $PCU=\frac{{TUA}_{i}}{Pop_{i}}$ where ${TUA}_{i}$ represents the total green space area within unit i, $Pop_{i}$ refers to the resident population within unit i.
**UGS quality**	Positive	Mean NDVI value of green spaces within each TAZ. $NDVI=\frac{NIR-RED}{NIR+RED}$ where NIR represents near-infrared reflectance and RED represents red-band reflectance.
**UGS service capacity**	Positive	The effective service amount provided by urban green spaces was assessed using the two-step floating catchment area (2SFCA) method, with TAZs serving as the basic analysis units. In this study, network-based travel distances were adopted to delineate service catchments so that accessibility reflects actual walking routes rather than straight-line Euclidean distance. A catchment radius of 500 m was applied, and a Gaussian distance-decay function was incorporated to capture the diminishing service contribution of green spaces as travel distance increases. $R_{j}=\frac{S_{j}}{\sum_{k\in \left\{d_{kj}\leq d_{\text{0}}\right\}}Pop_{k}}$, ${USC}_{j}=\sum_{j\in \{d_{ij}\leq d_{\text{0}}\}}R_{j}$ where $S_{j}$ is the area of green space unit j, and $\sum Pop_{k}$ is the total population within the catchment, $R_{j}$ is the supply ratio, ${USC}_{j}$ is the UGS service capacity for each TAZ unit i.

All indicators were normalized using min-max standardization to eliminate scale differences, as shown in Eq. (1):


$$S_{i}=\sum_{j=\text{1}}^{n}w_{j}\cdot X_{ij}^{\prime }$$ (1)

where $X_{i}^{\prime }$ is the normalized value of the indicator, $X_{i}$ is the original value, $X_{\text{max}}$ and $X_{\text{min}}$ are the maximum and minimum values of the indicator, respectively.

Subsequently, the entropy weight method was applied to determine the objective weights. The resulting weights were 0.22 for UGS service capacity, 0.21 for per capita UGS, 0.25 for UGS quality, and 0.32 for total UGS area, reflecting their relative contributions to the overall supply index. Based on these weights, a composite UGS supply index was constructed to represent the supply capacity of each spatial unit, as shown in Eq. (2):


$$S_{i}=\sum_{j=\text{1}}^{n}w_{j}\cdot X_{ij}^{\prime }$$ (2)

where $S_{i}$ denotes the supply index of the i-th evaluation unit, n is total number of indicators, $w_{j}$ is weight of the j-th indicator derived from the entropy weight method, $X_{ij}^{\prime }$ is standardized value of the j-th indicator for the i-th evaluation unit, and $\sum_{j=\text{1}}^{n}$ is summation over all indicators.

#### Evaluation of UGS demand.

On the demand side, the focus is on evaluating the green space services that residents can actually obtain and experience. Following the *China Standard for Urban Residential Area Planning and Design*, a 10-minute walking distance was adopted as the reasonable service radius of a basic residential unit, and buffers were constructed around residential points for analysis [21]. We delineated the 10-minute walkable area for each residential point using the Mapbox Isochrone API (https://docs.mapbox.com/playground/isochrone/), which generates network-based walkable service areas. The API simulates the area that can be reached within 10 minutes of walking along the actual road network and returns irregular polygon isochrones in GeoJSON format. These service areas were then converted into shapefiles and incorporated into the spatial analysis. Based on this framework, four indicators were selected to assess residents’ green space demand (Table 3). Accessible UGS coverage measures the proportion of residents who can access green space within a 10-minute walkable area, reflecting the breadth of service coverage and spatial equity [26]. Per capita accessible UGS further evaluates the amount of green space available to individuals within the accessible range, highlighting shortages in densely populated areas [27]. Accessible functional diversity, derived from POI data within the buffer, indicates the diversity of green space functions within residents’ daily activity spheres and the complexity of their needs [28]. Finally, average travel distance measures the cost of reaching the nearest green space, reflecting the convenience of actual use [29]. All spatial analyses of UGS demand were performed using ArcGIS Pro 3.3 to ensure accurate spatial processing and visualization.

**Table 3:** Demand-side indicators.

Indicator	Direction	Description
**Accessible UGS coverage**	Positive	Share of residents with access to UGS within a 10-minute walkable area. $AUC=\frac{Pop_{g}}{Pop_{tot}}$ where $Pop_{g}$ represents the number of residents who can access green space within 10-minute walkable area, and $Pop_{tot}$ is the total population of the study unit.
**Per capita accessible UGS**	Positive	Accessible UGS area per person in the walkable catchment. $PCAU=\frac{S_{g,acc}}{Pop_{acc}}$ where $S_{g,acc}$ is the total accessible green space area within the buffer zone of residential locations, and $Pop_{acc}$ denotes the population within the accessible range.
**Accessible functional diversity**	Positive	Diversity of facilities within the walkable area. $AFD=-\frac{\sum_{j=\text{1}}^{n}p_{j}\text{ln}\left(p_{j}\right)}{\text{ln}(n)}$ where $p_{j}$ denotes the proportion of facility type j within the buffer zone, and n is the total number of facility categories.
**Average travel distance**	Negative	Distance to the nearest UGS. $\begin{array}{c} ATD=\frac{\text{1}}{N}\sum_{i=\text{1}}^{N}d_{i} \end{array}$ where $p_{j}$ denotes the proportion of facility type j within the buffer zone, and n is the total number of facility categories.

During data processing, all demand-side indicators were normalized using min–max standardization, as shown in Eq. (1), to eliminate differences in scale. Subsequently, the entropy weight method was applied to determine the weights of each indicator. The resulting weights were 0.28 for per capita accessible UGS, 0.30 for average travel distance, 0.20 for accessible UGS coverage, and 0.22 for accessible functional diversity. Based on these weights, a composite UGS demand index was constructed to capture the actual level of residents’ demand for green space. The demand index was calculated as shown in Eq. (3):


$$D_{i}=\sum_{k=\text{1}}^{n}v_{k}\cdot Y_{ik}^{\prime }$$ (3)

where $D_{i}$ denotes the demand index of the i-th evaluation unit, n is total number of indicators, $v_{k}$ is weight of the k-th indicator derived from the entropy weight method, $Y_{ik}^{\prime }$ is standardized value of the k-th indicator for the i-th evaluation unit, and $\sum_{k=\text{1}}^{n}$ is summation over all indicators.

#### Measurement of supply–demand mismatch.

The imbalance between the supply and demand of UGS often carries inequitable characteristics, a phenomenon commonly referred to as supply–demand mismatch [30]. The essence of this mismatch lies in the disparity between the actual supply capacity of green spaces and the actual needs of residents: when supply is insufficient, it manifests as shortages, which may lead to compromised health and well-being, reduced recreational opportunities, and a decline in the quality of the living environment; whereas when supply exceeds demand, it manifests as surpluses, potentially resulting in inefficient resource utilization, increased maintenance costs, and diminished overall land-use efficiency [31]. To capture this unequal relationship, we introduced the concept of the supply–demand ratio and applied it to the assessment of UGS as the green space supply–demand ratio (GSDR) [32,14]. The calculation is given in Eq. (4):


$$GSDR=\frac{GS_{S}-GS_{D}}{\left(GS_{S}^{\text{max}}+GS_{D}^{\text{max}}\right)/\text{2}}\left\{ \;\begin{array}{c} >\text{0},surplus\\ =\text{0},balance\\ <\text{0},deficit \end{array}\right.\;$$ (4)

where GSDR represents the green space supply-demand ratio, used to quantify the degree of mismatch, $GS_{S}$ is the comprehensive supply index of green space in the study unit, $GS_{D}$ is the comprehensive demand index of green space in the study unit, $GS_{S}^{\text{max}}$ and $GS_{D}^{\text{max}}$ are the maximum values of the supply and demand indices within the study area, respectively.

When GSDR>0, it indicates that the green space supply of the unit exceeds demand, i.e., a surplus situation. When GSDR<0, it indicates that the green space supply is insufficient relative to demand, i.e., a deficit situation. When GSDR=0, it indicates that green space supply and demand are in a state of balance.

### Investigation of urban morphological influences on UGS supply–demand mismatch

#### Selection of urban morphology indicators.

The spatial mismatch between the supply and demand of UGS is often shaped by broader urban morphology and planning characteristics [33]. Different spatial structures not only influence the allocation of green spaces but also determine residents’ accessibility and the level of actual demand [34]. To identify these structural factors and their role in shaping mismatches, we introduces a set of urban morphology indicators to explain the spatial disparities in UGS supply–demand mismatch (Table 4). Specifically, road network density (RND) reflects travel accessibility. Areas with denser road networks generally exhibit higher green space accessibility and lower levels of mismatch, whereas sparse road networks may lead to the presence of green spaces that cannot be conveniently accessed, resulting in supply–demand imbalance [35]. Residential land proportion (RLP) indicates the intensity of residential activity and population concentration. A higher share of residential land often corresponds to greater demand; if green space supply is insufficient, unmet demand is more likely to occur [36]. Land use mix (LUM) captures the diversity of land functions. More complex functional patterns imply more diverse needs for green space services, and limited or homogeneous supply can lead to structural mismatches [28]. Floor area ratio (FAR) represents urban development density. High-intensity development tends to compress green space, reducing per capita availability and exacerbating mismatches [37]. Urban compactness index (UCI) reveals the overall spatial form of a city. Compact urban areas are more likely to achieve balanced green space distribution, while sprawling areas often face supply–demand separation due to low-density and dispersed development [38]. Finally, Public facilities coverage (PFC) reflects the level of investment in public services. Areas with insufficient public facility provision are often also underserved in terms of green space, meaning disadvantaged communities are at higher risk of experiencing green space shortages [39].

**Table 4:** Selected morphological driving factors of UGS supply–demand mismatch.

Indicator	Abb.	Description	Measurement
**Road network density**	RND	Measures the density of the road network within a unit, reflecting residents’ travel accessibility and potential green space service efficiency.	$RND_{i}=\frac{L_{i}}{A_{i}}$ where $L_{i}$ is the total length of roads within unit i, and $A_{i}$ is the area of unit i.
**Residential land proportion**	RLP	Represents the share of residential land within a unit, indicating the intensity of residential activities and potential demand for green space.	$RLR_{i}=\frac{S_{res,i}}{S_{tot,i}}$ where $S_{res,i}$ is the area of residential land within unit i, and $S_{tot,i}$ is the total area of unit i.
**Land use mix**	LUM	Captures the diversity of land use types using an entropy index, with higher values reflecting more complex functional demands for green space.	$H_{i}=-\frac{\sum_{j=\text{1}}^{n}p_{ij}\text{ln}\left(p_{ij}\right)}{\text{ln}(n)}$ where $p_{ij}$ represents the proportion of land use type j within unit i, and n is the total number of land use types.
**Floor area ratio**	FAR	Indicates the intensity of urban development, where higher values imply greater construction pressure and reduced green space availability.	$FAR_{i}=\frac{GFA_{i}}{S_{land,i}}$ where $GFA_{i}$ is the total gross floor area within unit i, and $S_{land,i}$ is the total area of construction land within unit i.
**Urban compactness index**	UCI	Reflects the geometric compactness of spatial units, with compact areas tending toward balanced green space allocation, while sprawling areas face higher mismatch risks.	$CI_{i}=\frac{\text{4}\pi A_{i}}{P_{i}^{\text{2}}}$ where $A_{i}$ is the area of unit i, and $P_{i}$ is the perimeter of unit i.
**Public facilities coverage**	PFC	Measures the proportion of land area covered by essential facility services, reflecting equity in public investment often aligned with green space provision.	$COV_{area}=\frac{S_{covered}}{S_{tot}}$ where $S_{covered}$ represents the area within the service radius of facilities (e.g., schools, hospitals), and $S_{tot}$ is the total area of the study unit.

#### Analysis of the driving factors using an XGBoost–SHAP model.

To further uncover the mechanisms influencing UGS supply–demand mismatch, we employs an XGBoost–SHAP framework to construct an interpretable machine learning regression model. XGBoost is an ensemble learning algorithm based on the gradient boosting framework, which performs well in handling nonlinear relationships, high-dimensional features, and complex feature interactions. As a tree-based algorithm, XGBoost exhibits inherent robustness to multicollinearity, as redundant predictors are naturally de-emphasized during the split selection process [17]. It has been widely applied in both regression and classification tasks. However, it should be noted that this model identifies associations rather than causal relationships, and thus the results reflect correlations instead of direct causation. XGBoost predicts outcomes using an additive tree model, expressed as Eq. (5):


$$\hat{y_{i}}=\sum_{t=\text{1}}^{T}f_{t}\left(x_{i}\right)$$ (5)

where ${\hat{y}}_{i}$ is the predicted value of the i-th sample, $f_{t}\left(x_{i}\right)$ represents the output of the t-th regression tree, and T is the total number of trees.

The model optimization objective combines a loss function and a regularization term, as shown in Eq. (6):


$$L=\sum_{i=\text{1}}^{n}l\left(y_{i},{\hat{y}}_{i}\right)+\sum_{t=\text{1}}^{T}\Omega \left(f_{t}\right)$$ (6)

where $l\left(y_{i},{\hat{y}}_{i}\right)$ is the loss function (e.g., mean squared error), and $\Omega \left(f_{t}\right)$ is the regularization term controlling tree complexity to prevent overfitting.

To ensure model robustness and generalization, a 5-fold cross-validation method was adopted. The dataset was randomly divided into five subsets, with four subsets used for training and one for validation in each iteration. The average performance across all folds was evaluated using three metrics: the coefficient of determination (R²), root mean square error (RMSE), and mean absolute error (MAE).

To enhance model interpretability, we adopted SHAP analysis on the XGBoost outputs. SHAP leverages the Shapley value concept from cooperative game theory to quantify each feature’s marginal contribution to predictions for individual samples [40]. The SHAP value $\phi_{i}$ for feature $x_{i}$ is calculated as Eq. (7):


$$\phi_{i}=\sum_{S\subseteq \{x_{\text{1}},\ldots ,x_{n}\}\backslash \{x_{i}\}}\frac{|S|!\left(n-|S|-\text{1}\right)!}{n!}\left[f\left(S\cup \{x_{i}\}\right)-f(S)\right]$$ (7)

where $\phi_{i}$ is the SHAP value of $x_{i}$, S denotes a subset of features excluding $x_{i}$, n is the total number of features, and $f\left(S\cup \{x_{i}\}\right)$ and f(S) are the model outputs with and without $x_{i}$, respectively.

## Results

### Spatial pattern of UGS supply–demand mismatch

#### UGS supply level.

**Fig 4:**
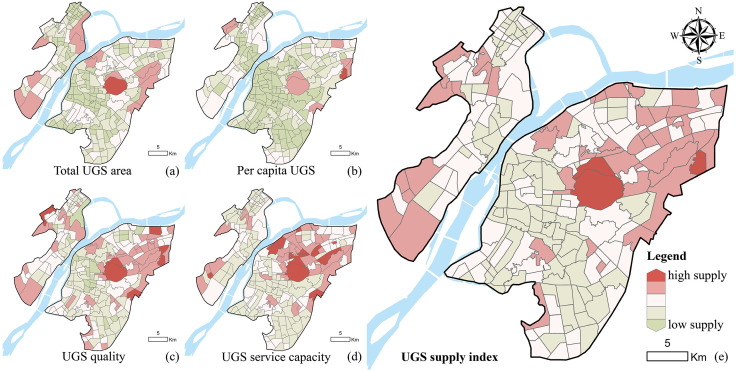
UGS supply level. **(a)** Total UGS area. **(b)** Per capita UGS. **(c)** UGS quality. **(d)** UGS service capacity. **(e)** UGS supply index. Base map and data from OpenStreetMap and OpenStreetMap Foundation.

The spatial distribution of UGS supply in the study area is shown in Fig 4. In terms of individual indicators, total UGS area and per capita UGS are relatively high in the central part of the city, while most other areas remain at lower levels. By contrast, UGS quality and UGS service capacity perform better in the northeastern streets, whereas the peripheral areas exhibit generally limited service capacity. Based on the integrated UGS supply index (Mean = 0.32, Std = 0.17, Max = 0.77, Min = 0.11), high-supply zones are primarily concentrated in the northeast and in suburban units containing large-scale or high-quality green spaces, notably in the vicinity of Xiaolingwei, Xigang, and Jiangpu subdistricts. In contrast, peripheral and riverside areas are characterized by more fragmented green resources, resulting in overall lower supply levels.

#### UGS demand level.

**Fig 5:**
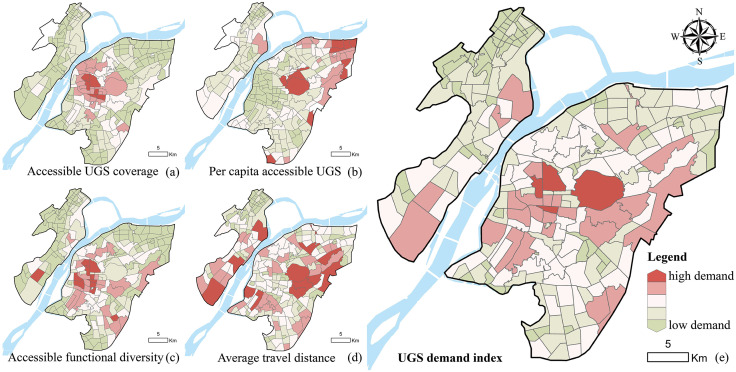
UGS demand level. **(a)** Accessible UGS coverage. **(b)** Per capita accessible UGS. **(c)** Accessible functional diversity. **(d)** Average travel distance. **(e)** UGS demand index. Base map and data from OpenStreetMap and OpenStreetMap Foundation.

The spatial distribution of UGS demand in the study area is illustrated in Fig 5. Regarding individual indicators, accessible UGS coverage is relatively low in the central neighborhoods, indicating higher demand, while the peripheral areas exhibit relatively higher coverage. Per capita accessible UGS is particularly low in the central and northeastern parts of the city, highlighting more pronounced demand in these areas. In the southern neighborhoods, Accessible functional diversity is insufficient, suggesting higher levels of demand. Results for average travel distance further indicate that a considerable number of high-demand neighborhoods exist, with a relatively scattered distribution. Based on the integrated UGS demand index (Mean = 0.17, Std = 0.16, Max = 0.51, Min = 0.02), significantly higher demand was observed in the central urban area and in certain neighborhoods along the eastern bank of the Yangtze River, particularly in Xiaolingwei, Xuanwumen, Hunan Road, and Wulaocun subdistricts, whereas demand in peripheral suburban areas was relatively low.

#### Characteristics of UGS supply–demand mismatch.

**Fig 6:**
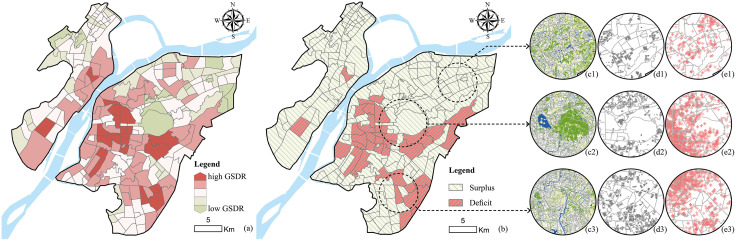
UGS supply–demand mismatch. **(a)** Green space supply–demand ratio (GSDR) values; **(b)** TAZs with surpluses and deficits; **(c)** Land use; **(d)** Residential distribution; **(e)** Public service facilities. Base map and data from OpenStreetMap and OpenStreetMap Foundation.

Significant spatial mismatches in UGS supply and demand were observed within the study area (Fig 6). Overall, no TAZ achieved a balanced state—each was either in surplus or deficit. Specifically, 203 TAZs exhibited green space surpluses (80.9%), with relatively abundant resources that could meet or even exceed residents’ needs; these were mainly distributed in peripheral areas. In contrast, 48 TAZs suffered from green space deficits (19.1%), largely concentrated in the central city and densely populated areas, where demand for green space was high but available land was limited. Comparative analysis of land use, residential distribution, and public service facility layouts revealed that supply gaps were primarily concentrated in residentially dense and facility-aggregated zones. These areas showed the most urgent demand yet frequently faced insufficient provision. By contrast, peripheral areas generally had lower residential density or larger-scale green spaces; while resources were abundant, spatial utilization efficiency remained relatively low.

### Interpretation of the effects of morphological factors on UGS supply–demand mismatch

The descriptive statistics of the core indicators used in the XGBoost analysis are presented in Table 5, which provides a systematic overview of the distributional characteristics of the variables. Five-fold cross-validation demonstrated strong predictive performance of the XGBoost model, with a mean R² of 0.86 and RMSE and MAE of 0.032 and 0.024, respectively.

**Table 5:** Distributional characteristics of core indicators.

Variable	Mean	SD	Min	Max	Median
**RND**	0.0089	0.0049	0.0000	0.0269	0.0086
**RLP**	0.1163	0.1243	0.0000	0.4614	0.0638
**LUM**	0.0942	0.0983	0.0000	0.3812	0.0699
**FAR**	0.6532	0.3438	0.0000	1.0000	0.7522
**UCI**	38.1820	31.4646	0.0001	139.8405	33.1294
**PFC**	0.6608	0.1490	0.2000	0.9069	0.6949
**Mismatch**	0.1187	0.1850	−0.6430	0.8349	0.1354

**Fig 7:**
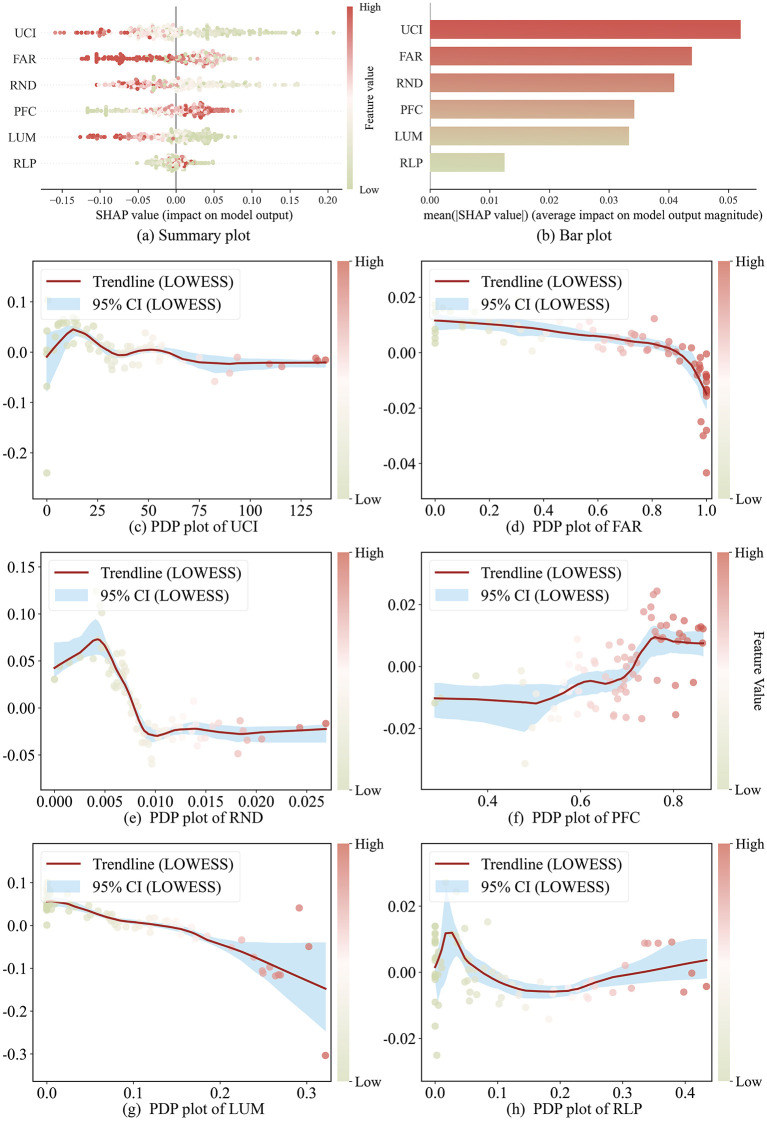
Importance and nonlinear effects of factors influencing UGS supply–demand mismatch based on XGBoost–SHAP analysis. **(a)** SHAP summary plot illustrating the impact of each feature on model output; **(b)** SHAP bar plot presenting mean absolute SHAP values, reflecting the relative importance of features; **(c–h)** Partial Dependence Plots (PDPs) depicting the nonlinear effects of different factors on UGS supply–demand mismatch, with LOWESS smoothing curves and 95% confidence intervals to highlight critical turning points.

Based on the XGBoost–SHAP analysis, the primary influencing factors, ranked by importance, were the UCI, FAR, RND, PFC, LUM, and RLP (Fig 7). Overall, UGS supply–demand mismatches are associated with a combination of urban form, development intensity, and population characteristics, with all factors exhibiting clear nonlinear effects and critical turning points. Among these, UCI and FAR showed the strongest contributions, indicating not causation but a pattern in which compact urban form and development intensity are statistically linked with variations in mismatch levels. Specifically, UCI exerted a positive influence at lower levels but shifted to a negative pattern once it exceeded a threshold of approximately 25, suggesting an association where moderate compactness corresponds to lower mismatch, whereas higher compactness corresponds to higher mismatch. FAR displayed a consistently negative contribution, with a marked decline occurring beyond approximately 0.65, reflecting a statistical relationship in which higher development intensity is linked to more pronounced mismatch. RND contributed to reducing mismatches at lower levels, but its effect weakened and eventually turned negative after reaching approximately 0.01, showing a non-linear association where dense road networks coincide with reduced green space availability. PFC exhibited an overall positive influence, with an enhanced contribution emerging once it surpassed approximately 0.70, indicating that areas with greater public facility coverage tend to show lower mismatch. LUM was negatively related to mismatch, suggesting that higher land-use diversity is associated with increased competition for limited land resources. Finally, RLP had little influence at lower levels but began to show a pronounced increase in mismatch once it exceeded approximately 0.12, indicating a statistical link between higher residential land proportion and increased demand pressure.

## Discussion

### Key contributions of the research

This study makes several key contributions. Theoretically, this study moves beyond the traditional focus on either supply or demand alone by developing a scalable integrated assessment framework that provides a more comprehensive basis for identifying UGS supply–demand mismatches. In addition, using an interpretable machine-learning approach, the study uncovers the nonlinear relationships and key thresholds linking urban morphology to UGS mismatches, revealing mechanisms that traditional linear, single-indicator methods overlook. Practically, we propose targeted and spatially differentiated planning strategies for UGS systems. Unlike one-size-fits-all greening recommendations, our findings support context-specific interventions that offer planners a feasible pathway to convert “potential supply” into effective and equitable provision.

### Advancing the understanding of spatial mismatch and inequity in UGS supply–demand

Regarding Research Question 1, our findings reveal a pronounced spatial mismatch between UGS supply and demand within the central urban area of Nanjing. Specifically, 48 TAZs experienced deficits, while 203 TAZs exhibited surpluses, forming a typical spatial pattern of “shortages in the core and surpluses in the periphery”. Similar spatial imbalances have also been observed by other researchers in high-density cities such as Guangzhou, New York, and Seoul, where compact development and concentrated populations exacerbate disparities in green space accessibility and service levels [41]. This imbalance highlights unequal access to green spaces among residents and reflects the misalignment between green space distribution and population concentration under rapid urbanization [42]. The emergence of this inequitable pattern is primarily driven by the interplay of high population density, intensive land use, and institutional constraints in the urban core [43]. On the one hand, fragmented land parcels, complex property rights, high redevelopment costs, and floor area ratios already close to regulatory limits leave little room for adding or upgrading green spaces [44]. Moreover, historic districts and the concentration of multiple urban functions place green space at a disadvantage when competing with residential, commercial, and transport land uses [45]. On the other hand, although peripheral areas contain abundant green spaces, they often suffer from functional homogeneity, low-density development, inadequate public transport, and poor road connectivity. Together with insufficient supporting facilities, these conditions hinder the integration of green spaces into daily life, resulting in a situation where “quantitative abundance” fails to translate into “qualitative accessibility”. Collectively, these factors reinforce the entrenched pattern of “core shortages–peripheral surpluses”. This pattern not only reflects contradictions in spatial structure but also reveals inefficiencies in resource utilization: green spaces are most needed in the core but lack expansion capacity, while peripheral green spaces remain underutilized due to limited accessibility and functionality [46]. Consequently, even when overall green space provision reaches an adequate level, residents’ actual benefits and experiences of use may still fall short.

More importantly, the mismatch is amplified by socio-economic disparities [47]. The urban core accommodates a high proportion of vulnerable groups—such as low-income residents, the elderly, and migrant workers—who are heavily reliant on walkable green spaces yet reside in areas of greatest scarcity [48]. In contrast, the resource advantages of peripheral areas tend to benefit groups with better transport access and more dispersed living environments, often including middle- and high-income households and newly developed residential communities. This aligns with recent findings that socioeconomic stratification significantly affects environmental equity, with disadvantaged populations often located in green-deficient zones [49]. This indicates that UGS mismatches constitute an ecological and spatial governance challenge as well as a critical concern for social equity [50]. Therefore, future urban planning should move beyond a purely “area-oriented” approach and place greater emphasis on aligning the service capacity of green spaces with residents’ needs, promoting equitable and balanced UGS provision through coordinated improvements on both the supply and demand sides.

### Implications for improving the balance of UGS supply and demand

With respect to Research Question 2, the results indicate that UGS supply–demand mismatch is shaped by multiple factors, with UCI and FAR contributing most significantly. This suggests that compact urban forms and high-intensity development are key drivers exacerbating the imbalance between supply and demand. This finding aligns with previous studies, which emphasize that moderate compactness facilitates quicker access to green spaces, whereas excessive compactness and intensive development often result in insufficient green space [51]. Such conditions are widely observed in many large cities (e.g., Beijing, Shanghai), where high-density development and population concentration create acute shortages of UGS [51]. In addition, The negative effect of RND can be explained by the fact that denser road networks occupy substantial urban land and fragment green space patches, thereby reducing overall supply and exacerbating supply–demand mismatch [52]. Recent studies have similarly found that increased road-network density in highly compact urban areas tends to amplify green-space fragmentation and reduce accessibility [53]. However, in some medium-sized cities with more balanced land-use structures, higher road density may instead enhance accessibility to distributed green areas, highlighting the contextual variability of this relationship [54]. The positive effect of PFC indicates that areas with well-developed educational and healthcare facilities often coincide with more systematic investments in public space, promoting more rational green space layouts and alleviating local mismatches. Yet, in densely populated districts, the combined concentration of population and public facilities intensifies demand pressures [55]. LUM shows a negative association with balance, suggesting that while functional diversity enhances urban vitality, under high-density development limited land is more intensively contested by competing uses, often at the expense of green space [28]. Finally, RLP highlights that areas with high population density tend to face the greatest green space pressure, as rapidly growing demand collides with limited expansion of supply, thereby aggravating mismatches [56]. This pattern is consistent with recent studies on high-density urban environments, which similarly reported that population agglomeration and compact development increase the likelihood of low-supply–high-demand clusters of UGS and thus intensify spatial inequities in green access [57,58].

Based on these findings, we propose broadly applicable planning strategies aimed at alleviating UGS supply–demand mismatch. In high-density built-up areas, where demand is intense but supply is insufficient, the focus should be on intensifying the reuse and micro-renewal of existing urban space, for example, through pocket parks, rooftop greening, and street-level greenery, thereby increasing accessible green space and improving residents’ actual benefits [59]. However, the implementation of such micro-renewal projects often faces several practical challenges, including fragmented land ownership, limited financial investment, and varying levels of resident participation [60]. These obstacles can restrict project continuity and scalability, especially in older neighborhoods with complex property rights. To address these issues, multi-stakeholder collaboration mechanisms should be established, encouraging joint participation from local governments, community organizations, and private enterprises. Furthermore, introducing flexible land-use policies, diversified funding models, and community co-governance mechanisms could help enhance feasibility and long-term sustainability [61]. In districts with dense road networks, the balance between transportation infrastructure and green space should be optimized to prevent over-expansion of roads from further reducing green space availability [62]. In densely populated neighborhoods with strong demand pressures, integrated planning of public service facilities and multifunctional green spaces is essential to enhance the fit between supply and demand while simultaneously providing ecological, recreational, and service benefits [63]. In peripheral areas, where green space resources are relatively abundant but underutilized, efforts should focus on improving transportation connections and supportive facilities to shorten travel distances and enhance accessibility, thus converting “potential supply” into “effective supply” [64]. At the same time, participatory design and social inclusion should be emphasized to ensure that different groups—particularly low-income and elderly residents—can benefit equitably from green space improvements [65,66]. Through these multidimensional strategies, it is possible to expand service capacity on the supply side while improving accessibility and user experience on the demand side, thereby promoting a more balanced and equitable distribution of UGS and offering valuable insights for sustainable urban planning.

### Limitations and future research

This study still has certain limitations that warrant improvement and expansion in future research. First, the analysis of UGS supply and demand relies on static data, which limits the ability to capture dynamic changes and long-term trends under rapid urban development. Future studies could integrate multi-temporal remote sensing imagery and time-series statistical data to conduct dynamic monitoring and evolution analyses. Second, although the UGS supply and demand indicators used in this study cover the main dimensions, they may not fully represent all relevant factors influencing urban green space services. Future work could refine the indicator system to incorporate a wider range of influencing factors and better reflect the complexity of green space functions. Third, while the XGBoost–SHAP model provides valuable interpretability by quantifying the contribution and direction of each factor, it remains a data-driven and correlational approach. The analysis identifies associations rather than causal relationships. Future studies could integrate causal inference techniques or spatial econometric models to further validate and deepen the understanding of the underlying influencing mechanisms. Finally, although the present research emphasizes objective spatial analysis, it does not fully integrate residents’ subjective perceptions, usage preferences, or cultural differences as social–psychological factors. Future work could address this gap by incorporating perceived equity evaluations through questionnaire surveys, social media data, and other participatory approaches.

## Conclusion

This study quantified the supply, demand, and mismatches of UGS in the central area of Nanjing, revealing their spatial imbalances and inequities. By employing an interpretable XGBoost–SHAP model, we identified the nonlinear effects of urban morphology on UGS supply–demand mismatch, thereby overcoming the limitations of traditional linear models in capturing complex spatial mechanisms. The results show that the central districts of Nanjing face a pronounced imbalance: 48 TAZs experience a deficit, while 203 TAZs show a surplus, reflecting a pattern of shortage in the urban core and surplus at the periphery. This imbalance is primarily driven by multiple urban morphological factors. UCI and FAR exert the strongest negative influences, indicating that overly compact and high-intensity development aggravates green space shortages. RND and LUM also contribute negatively by fragmenting green patches and increasing land-use competition. In contrast, PFC and RLP play a positive role in mitigating mismatches by improving spatial organization and service accessibility. Based on these findings, we proposed targeted and practical strategies to mitigate UGS supply–demand mismatches. In high-density built-up areas, reusing existing spaces through pocket parks, rooftop greening, and small-scale micro-renewal can help alleviate shortages. In areas with fragmented ownership or financial constraints, multi-stakeholder collaboration and flexible land-use policies can enhance project feasibility. In peripheral districts, improving transport connectivity and supportive facilities is essential to convert “potential supply” into “effective provision”. These measures contribute to equitable urban planning.
